# Cardiovascular Safety Profile of BRAF and MEK Inhibitors in Melanoma: FAERS Data Through a Retrospective Disproportionality Analysis (2014–2023)

**DOI:** 10.3390/cancers17111755

**Published:** 2025-05-23

**Authors:** Maria Antonietta Barbieri, Giulia Russo, Giuseppe Cicala, Concetta Zito, Edoardo Spina, Nicola Silvestris, Mariacarmela Santarpia

**Affiliations:** 1 Department of Clinical and Experimental Medicine, University of Messina, 98125 Messina, Italy; mariaantonietta.barbieri@unime.it (M.A.B.); giuliarusso.ab@gmail.com (G.R.); giuseppe.cicala@unime.it (G.C.); concetta.zito@unime.it (C.Z.); edoardo.spina@unime.it (E.S.); 2 Medical Oncology Department, IRCCS Istituto Tumori “Giovanni Paolo II”, 70124 Bari, Italy; 3 Medical Oncology Unit, Department of Human Pathology in Adulthood and Childhood “Gaetano Barresi”, University of Messina, 98125 Messina, Italy; mariacarmela.santarpia@unime.it

**Keywords:** cardiovascular toxicity, disproportionality analysis, BRAF inhibitors, FAERS, MEK inhibitors, melanoma

## Abstract

**Introduction:** The combination of BRAF and MEK inhibitors (BRAF/MEKi) has significantly improved survival in melanoma patients with BRAF V600 mutations. However, these agents can cause cardiovascular (CV) toxicity, compromising efficacy. This study evaluated the CV adverse events (cAEs) associated with BRAF/MEKi using the U.S. FDA Adverse Event Reporting System (FAERS) to identify new signals of disproportionate reporting (SDRs). **Methods:** Descriptive and disproportionality analyses were conducted on reports listing dabrafenib (D), vemurafenib (V), encorafenib (E), trametinib (T), cobimetinib (C), or binimetinib (B) as suspects in monotherapy or combination therapy (D + T, V + C, E + B), with melanoma as the indication and at least one cAE. Standardized MedDRA Queries (SMQs) related to cAEs, including bradyarrhythmias and tachyarrhythmias, cardiac failure, cardiomyopathy, thrombotic events, ischaemic heart disease, and myocarditis/pericarditis, were analyzed. **Results:** Of the 14,077,067 reports retrieved, 18,370 (0.1%) were linked to BRAF/MEKi, with 1591 (8.7%) reporting cAEs, primarily in combination therapy (*n* = 1268). Disproportionality analysis identified 64 clinically relevant SDRs, most of which were unexpected. Notable findings included bradyarrhythmias, such as QT prolongation with D + T (*n* = 59; Reporting Odds Ratio, ROR = 5.09, 95% Confidence Interval, CI = 3.94–6.58), cardiac failure with V + C (29; 3.76, 2.6–5.42), and tachyarrhythmias, particularly atrial fibrillation with D + T (99; 2.37, 1.94–2.89). Among embolic and thrombotic events, clinically significant SDRs were observed for disseminated intravascular coagulation with D + T (38; 10.22, 7.42–14.06) and pulmonary embolism with V + C (22; 2.79, 1.83–4.24). **Conclusions:** Our findings underscore the need for comprehensive CV monitoring in patients receiving BRAF/MEKi therapy to prevent or detect cAEs early and reduce treatment-related risks, particularly in high-risk populations.

## Simple Summary

BRAF and MEK inhibitor combinations (BRAF/MEKi) have significantly improved survival in melanoma patients with BRAF mutations, but can induce a wide spectrum of cardiovascular (CV) toxicities. This study evaluated the CV adverse events (cAEs) associated with BRAF/MEKi using the U.S. FDA Adverse Event Reporting System to identify new signals of disproportionate reporting (SDRs). Of the 14,077,067 reports retrieved, 18,370 (0.1%) were linked to BRAF/MEKi, with 1591 (8.7%) reporting cAEs, primarily in combination therapy. Sixty-four clinically relevant SDRs were identified, most of which were unexpected, including bradyarrhythmias such as QT prolongation, atrial fibrillation and disseminated intravascular coagulation with D + T, cardiac failure and pulmonary embolism with V + C. Comprehensive CV monitoring in patients receiving BRAF/MEKi therapy is fundamental to prevent or detect cAEs early and reduce treatment-related risks, particularly in high-risk populations.

## 1. Introduction

Melanoma is the most aggressive form of skin cancer resulting from the malignant transformation of melanocytes. In 2022, there were an estimated 331,647 new cases of melanoma of the skin, accounting for 1.7% of global cancer diagnoses, and 58,645 deaths [1].

During the last decade, several therapeutic approaches, including targeted therapy and immunotherapy, have been developed and approved for clinical use, leading to remarkable improvements in the long-term survival of melanoma patients [2]. BRAF is one of the most frequently mutated oncogenes found in cutaneous melanoma, present in 40–50% of cases [3]. The most frequent mutation involves codon 600, which is located within the kinase domain. The V600E, accounting for 70–88% of cases, is a missense mutation causing a substitution of a valine with glutamic acid. The second most common variant is V600K, consisting of the substitution of valine with lysine. Oncogenic mutations lead to constitutive activation of the BRAF/MEK/ERK (MAPK) signaling pathway, which plays a key role in the development and progression of melanoma by regulating a wide range of cellular processes, including proliferation, survival, invasion and metastasis, as well as by modulating immune responses [4,5,6]. Specific tyrosine kinase inhibitors (TKIs), including vemurafenib (V), dabrafenib (D) and encorafenib (E), have been developed to target melanoma with BRAF V600 mutations. These drugs specifically bind the ATP-binding site of BRAF-mutated proteins, thereby inhibiting their activity and leading to tumor regression. However, despite initial significant responses, the use of BRAF inhibitors (BRAFi) has been associated with the development of resistance due to the paradoxical activation of the MEK-mediated signaling cascade. Moreover, an increased incidence of secondary skin tumors has been observed. These limitations have been addressed by combining these agents with inhibitors of MEK [7]. In large clinical trials, different combinations of BRAF- and MEK inhibitors (BRAF/MEKi), including vemurafenib + cobimetinib (V + C), dabrafenib + trametinib (D + T), and encorafenib + binimetinib (E + B), have widely demonstrated improvements in clinical outcomes over the single agent BRAFi, with a manageable safety profile [8,9,10,11,12,13]. Currently, these combinations are used in clinical practice for BRAFV600-mutant melanoma patients with advanced/metastatic disease or in the adjuvant setting [2,14]. The different combinations are associated with a variable incidence of side effects [15]. Most toxicities are manageable, reversible, and rarely lead to treatment discontinuation [16]. Given the high effectiveness of these drugs, it is of huge importance to recognize and promptly manage the onset of BRAF/MEKi-related adverse events (AEs). As long-term data continue to demonstrate sustained survival benefits in a significant proportion of patients, and considering the increasing prevalence of hypertension and other cardiovascular (CV) comorbidities in the general population, particular attention should be given to the risk of potentially life-threatening toxicities, such as CV AEs (cAEs). Some cAEs induced by BRAF/MEKi have been observed, including left ventricular systolic dysfunction (LVSD), hypertension, QT interval prolongation, atrial arrhythmia, and venous thromboembolism [17,18,19]. Although cAEs have been documented in registration trials and meta-analyses [15,17,18,19], it is important to note that patients with clinically significant CV disease were excluded from the primary studies, suggesting that the true incidence of these AEs may be underestimated. In addition, previously undetected cAEs may not have emerged in randomized clinical trials due to limited sample sizes and strict inclusion criteria. Post-marketing pharmacovigilance studies relying on spontaneous reporting systems (SRS) have proven their importance as tools for obtaining a more comprehensive understanding of these medications’ safety profiles, especially for identifying AES that may not have been observed in randomized controlled trials [20,21,22].

Building on this premise, the present study investigates whether treatment with BRAF/MEKi in melanoma may be associated with previously unrecognized cAEs. By leveraging the U.S. Food and Drug Administration Adverse Event Reporting System (FAERS) database, we evaluated the presence of potential signals of disproportionate reporting (SDRs) related to cAEs and sought to characterize their clinical features in real-world settings.

## 2. Materials and Methods

### 2.1. Study Design

A retrospective pharmacovigilance study was conducted to analyze reports of cAEs linked to BRAFi or MEKi as monotherapy or the combination of a BRAF/MEKi approved for melanoma. This analysis utilized the FAERS database, employing both descriptive and disproportionality methods. FAERS is a widely used public resource containing over 20 million reports submitted by patients, healthcare professionals, and pharmaceutical companies from the United States, Europe, and Asia. Detailed characteristics of this database have been described in prior studies [21,23].

### 2.2. Data Processing and Duplicate Report Management

Reports spanning from the first quarter of 2014 (Q1) to the fourth quarter of 2023 (Q4) were downloaded from the zipped ASCII FAERS quarterly data extract files, available at https://fis.fda.gov/extensions/FPD-QDE-FAERS/FPD-QDE-FAERS.html (accessed on 31 October 2024). This timeframe aligns with the year of approval for the first combination of BRAF/MEKi for melanoma. Data were extracted from the DEMO, DRUG, INDI, OUTC, REAC, and THER files. To create a comprehensive dataset, the INDI and THER files were integrated with the DRUG data, resulting in a combined file labeled combined DRUG. Similarly, OUTC data were merged with DEMO data to form the combined DEMO file, while REAC data were directly utilized to generate the combined REAC file. All datasets were meticulously cleaned by removing duplicate entries based on a two-step deduplication process to ensure data integrity. Initially, duplicate entries were identified and removed based on primary and case ID codes. In instances where multiple case IDs were associated with the same primary ID, only the most recent version was retained, following FDA guidelines. A second deduplication step was then conducted by evaluating case similarity across key variables such as the AE type, onset date, gender, age, reporting country, and suspected drug. For duplicate reports sharing the same primary ID, only the most recent version of the case ID was retained, adhering to FDA guidelines. Further exclusions were applied: premarketing reports supported by literature references were removed from the combined DEMO file, and reports involving at least one investigational product, investigational biosimilar, unspecified ingredient, or blinded product were excluded from the combined DRUG file. Additionally, cases with the Preferred Term (PT) “no adverse event” were excluded from the combined REAC file.

### 2.3. Case Selection

Reports listing D, T, V, C, E, or B as the primary suspect (PS) were identified and categorized as “cases” of monotherapy. Additionally, reports in which one of these drugs was listed as the PS and the corresponding drug from an approved BRAF/MEKi combination was listed as the secondary suspect (SS) were also included as “cases” of combination. These combinations specifically included D + T, V + C, and E + B. To minimize therapeutic biases, reports indicating as “indi_pt” conditions other than melanoma were excluded. Among the previously defined cases, those with at least one AE coded under a PT from the narrow version of specific Standardized MedDRA Queries (SMQs) in MedDRA^®^ version 26.1 were identified as cAE cases. The selected SMQs included the following: “Bradyarrhythmias (incl conduction defects and disorders of sinus node function)”, “Cardiac failure”, “Cardiomyopathy”, “Embolic and thrombotic events”, “Ischaemic heart disease”, “Noninfectious myocarditis/pericarditis”, and “Tachyarrhythmias (incl supraventricular and ventricular tachyarrhythmias)”. A comprehensive list of all included PTs is available in Supplementary Table S1.

### 2.4. Data Analyses

A descriptive statistical analysis of demographic and clinical characteristics was performed to evaluate all AEs and cAEs for monotherapy and a combination of BRAF/MEKi. Continuous variables were summarized as medians with interquartile ranges (Q1–Q3), while categorical variables were presented as absolute counts and corresponding percentages. The time-to-onset (TTO) of cAEs was calculated as the number of days between the drug initiation date and the event date, and it was expressed as a median (Q1–Q3) through a box plot for each drug or combination of interest.

A disproportionality analysis was conducted at the PT level for all the above-mentioned SMQs to detect SDRs for drug–event pairs using a case/non-case methodology. This method compared the proportion of reports with a specific cAE linked to a particular drug (cases) to the proportion of reports with the same cAE associated with all other drugs in the FAERS database (non-cases), which served as the reference group. The reporting odds ratio (ROR) and its 95% confidence interval (CI) were calculated to quantify the association. A SDR was identified when the lower limit of the 95% CI for the ROR exceeded 1, if there were at least three reports for the drug–event combination. AEs not included in the FDA Full Prescribing Information under the Adverse Reactions section at the time of the study were considered unexpected. To assess clinical relevance, the analysis utilized the European Medicines Agency’s (EMA) lists of Important Medical Events (IMEs, version 27.1) and Designated Medical Events (DMEs), which include rare but serious events likely to be drug-induced. Statistical significance was set at *p* < 0.05. Data processing and analyses were performed using R Studio (Posit Software, PBC, Boston, MA, USA; version 4.4.1). The study adhered to the guidelines outlined in the “REporting of A Disproportionality analysis for drUg Safety signal detection using individual case safety reports in PharmacoVigilance” (READUS-PV) statement [24].

## 3. Results

### 3.1. Cases Selection

**Figure 1:**
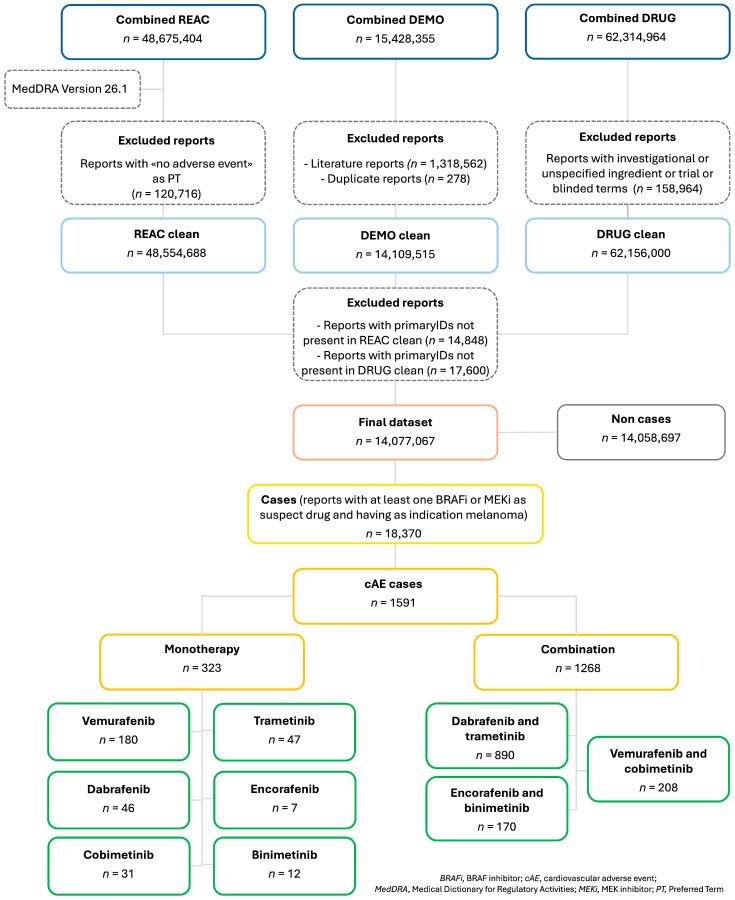
Flowchart of report selection process in FAERS database.

During the study period, a total of 14,077,067 reports were retrieved after merging the ASCII files and applying the case selection criteria outlined in the Materials and Methods section. From the final dataset, 18,370 reports (0.1%) were found to be related to our drugs of interest, with melanoma as the indication. Among these, 8895 reports (48.4%) were related to D + T, 3097 (16.9%) to E + B, 2174 (11.8%) to V, 2111 (11.5%) to V + C, 852 (4.6%) to D, 695 (3.8%) to T, 263 (1.4%) to C, 161 (0.8%) to B, and 122 (0.7%) to E. Of the total cases, 1591 (8.7%) reports were classified as cAEs, with the highest proportion reported for the BRAF/MEKi combination (1268 cases), while only a minority, at 323, were linked to BRAFi monotherapies. In detail, 890 cases were reported for D + T (55.9%), 208 for V + C (13.1%), 170 for E + B (10.7%), and 180 for V (11.3%). Lower proportions of cAE cases were reported for BRAFi and MEKi used as monotherapy (Figure 1).

### 3.2. Descriptive Analysis

The characteristics of reports related to all AEs and cAEs for BRAF/MEKi, both in combination and monotherapy, are summarized in Table 1. Reports of cAEs were generally linked to elderly people more than any AEs, with a median (Q1–Q3) age of 66 (55–74) years for combination therapy and 65 (58–74) years for monotherapy.

CAE-related reports also presented a higher frequency of elderly patients, accounting for 43.3% of BRAF/MEKi combination therapy reports and 45.2% of BRAFi reports. Among individual treatments, V + C-related reports had the highest proportion of elderly people with cAEs (*n* = 119; 57.2%) (Table S2), while E and C also showed a notable percentage (*n* = 4; 57.1% and *n* = 16; 51.6%) (Table S3). CAE-related reports generally involved male patients more frequently than female ones. However, an exception was observed with E and T, where the proportion of females with cAEs was higher than males (*n* = 5; 71.4% and *n* = 25; 53.2%, respectively) (Table S3). Physicians reported a greater percentage of cAEs in monotherapy (*n* = 225; 69.7%) compared to combination therapy (*n* = 692; 54.6%). In contrast, consumer-reported cAEs were lower in monotherapy (*n* = 35; 10.8%) than in combination therapy (*n* = 235; 18.5%).

Hospitalization rates and other serious (IME) AEs were notably higher among cAEs compared to all AEs, with monotherapy exhibiting a greater hospitalization burden (*n* = 149; 46.1%), particularly for C (*n* = 19; 61.3%) and D (*n* = 25; 54.3%) (Table S3). Conversely, combination therapy had a higher onset of other serious (IME) cAEs (*n* = 602; 47.5%), especially with E + B (*n* = 89; 52.4%) and D + T (*n* = 439; 49.3%) (Table S2). Regarding temporal trends, the highest reporting year for combination therapy cAEs was 2019 (*n* = 209; 16.5%), driven primarily by V + C (*n* = 45; 21.6%). In contrast, monotherapy cAEs peaked earlier, between 2015 and 2016, especially for V in 2015 (*n* = 59; 32.8%) and D in 2016 (*n* = 13; 28.3%).

### 3.3. Cardiovascular Adverse Events

**Figure 2:**
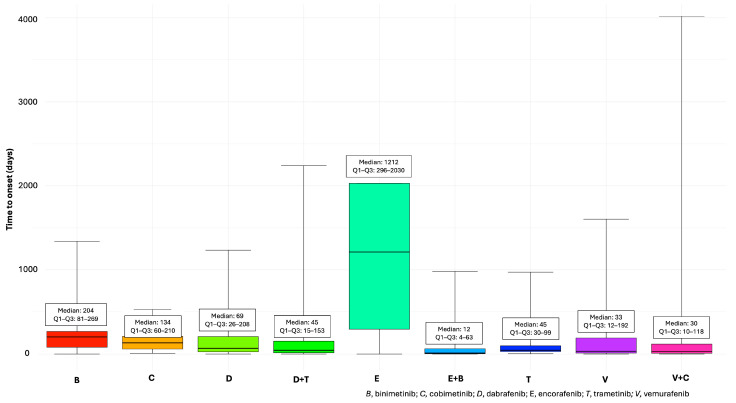
Time to onset of cardiovascular adverse events.

Considering the TTO of cAEs, combination therapies tended to have an earlier onset of cAEs, with E + B showing the shortest median (Q1–Q3) TTO with 12 (4–63) days, followed by V + C (30 days, Q1–Q3: 10–118). In contrast, monotherapies exhibited greater variability in cAE onset, with some drugs showing delayed TTO. Notably, E monotherapy stood out with a median (Q1–Q3) TTO of 1212 (296–2030) days. On the other hand, T (45 days, Q1–Q3: 30–99) and V (33 days, Q1–Q3: 12–192) demonstrated relatively shorter onset times, aligning more closely with combination therapy trends (Figure 2).

**Figure 3:**
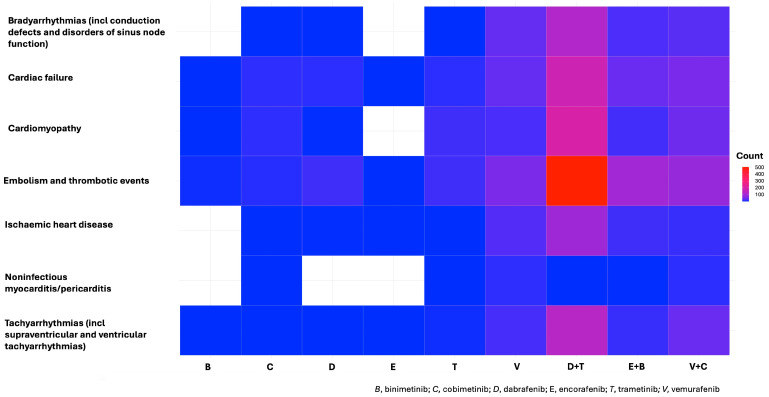
Number of cardiovascular adverse events identified by each drug in monotherapy or combination therapy, based on the selected Standardized MedDRA Queries (SMQs) within the Medical Dictionary for Regulatory Activities (MedDRA^®^) (version 26.1).

A total of 2148 cAEs were identified across 1591 reports, based on the selected SMQs. Embolic and thrombotic events were the most frequently reported (*n* = 807; 50.7%), followed by cardiac failure (*n* = 351; 22.1%) and cardiomyopathy (*n* = 329; 20.7%). Bradyarrhythmias (incl conduction defects and disorders of sinus node function) and tachyarrhythmias (incl supraventricular and ventricular tachyarrhythmias) showed a similar reporting trend (*n* = 227; 14.3% and *n* = 226; 14.2%, respectively). Ischaemic heart diseases were reported in 170 cases (10.7%), while noninfectious myocarditis/pericarditis was less commonly observed, with only 38 cases (2.4%). Among combination therapies, D + T exhibited the highest number of cAEs, particularly for embolic and thrombotic events (*n* = 509), cardiomyopathy (*n* = 201), and cardiac failure (*n* = 175). Among monotherapies, V was linked to the highest number of cAEs, particularly embolic and thrombotic events (*n* = 56), cardiac failure and bradyarrhythmias (incl conduction defects and disorders of sinus node function) (both *n* = 39) (Figure 3).

### 3.4. Disproportionality Analysis

A total of 100 SDRs were identified for PTs associated with the SMQs of interest in BRAFi and MEKi, both as monotherapy and combination therapy, with 64 of these being clinically relevant, as reported in the IME list. Most of these SDRs were not documented in the current drug labels. For bradyarrhythmias (incl conduction defects and disorders of sinus node function), unexpected cAEs with clinical relevance were observed with D + T, including Brugada syndrome (*n* = 4; ROR = 25.12, 95% CI = 9.35–67.45), electrocardiogram QRS complex prolonged (5; 6.53, 2.71–15.73), electrocardiogram QT prolonged (59; 5.09, 3.94–6.58), and electrocardiogram repolarization abnormality (8; 32.89, 16.33–66.27) (Table 2). In monotherapy, both D and C showed unexpected clinically relevant SDRs for electrocardiogram QT prolonged (D: 5; 4.49, 1.86–10.82, C: 3; 8.78, 2.81–27.4) (Table S4).

Regarding cardiac failure, unexpected SDRs were observed with V + C, including cardiac failure (29; 3.76, 2.6–5.42) and left ventricular failure (10; 32.56, 17.47–60.7) (Table 2). E + B was associated with cardiogenic shock (5; 3.91, 1.63–9.4) (Table 2), while C was also linked to cardiac failure (4; 4.16, 1.55–11.17) and cardiac failure acute (4; 58.11, 21.64–156.09) (Table S4). Considering cardiomyopathy, D + T demonstrated an unexpected clinically relevant SDR for cardiotoxicity (7; 4.22, 2.01–8.87) (Table 2). In monotherapy, V showed unexpected SDRs with clinical relevance for cardiotoxicity (6; 14.84, 6.65–33.1) and hypertensive cardiomyopathy (3; 127.96, 40.78–401.49), while T was linked to cardiotoxicity (4; 31.02, 11.6–82.96) (Table S4). Among tachyarrhythmias (incl supraventricular and ventricular tachyarrhythmias), D + T was associated with unexpected SDRs with clinical relevance for atrial fibrillation (AF) (*n* = 99; ROR = 2.37, 95% CI = 1.94–2.89) and ventricular arrhythmia (4; 4.13, 1.55–11.01) (Table 2), while in monotherapy, T was related to AF (7; 2.14, 1.02–4.51) (Table S4). Considering noninfectious myocarditis/pericarditis, V + C was associated with an unexpected SDR for myocarditis (*n* = 10; ROR = 10.26, 95% CI = 5.51–19.1), as well as E + B (6; 4.18, 1.88–9.32). Although D + T also reported cases of myocarditis (*n* = 4), no SDR was detected. For monotherapy, only V showed an unexpected SDR for pericarditis (12; 6.71, 3.8–11.84).

Focusing on unexpected SDRs related to ischaemic heart disease, D + T was associated with unexpected SDRs with clinical relevance for coronary artery stenosis (*n* = 10; ROR = 7.28, 95% CI = 3.91–13.55) (Table 2). In monotherapy, V displayed an unexpected clinically relevant SDR for acute coronary syndrome (3; 4.24, 1.37–13.16) (Table S4). Several unexpected SDRs with clinical relevance were identified for embolic and thrombotic events: D + T was associated with disseminated intravascular coagulation (DIC) (*n* = 38; ROR = 10.22, 95% CI = 7.42–14.06), splenic infarction (9; 15.78, 8.18–30.43), cerebral ischaemia (12; 7.24, 4.11–12.77), hemiparesis (29; 4.42, 3.07–6.37), hemiplegia (10; 3.06, 1.64–5.68), paraparesis (7; 11.1, 5.28–23.35), paraplegia (9; 8.47, 4.4–16.3), and quadriplegia (3; 4.84, 1.56–15.04). V + C was linked to cerebral ischaemia (8; 20.37, 10.16–40.83), pulmonary embolism (22; 2.79, 1.83–4.24), and deep vein thrombosis (10; 1.91, 1.03–3.55). E + B displayed unexpected SDRs with clinical relevance for DIC (4; 3.06, 1.15–8.17), blindness transient (5; 4.78, 1.99–11.49), hemiparesis (7; 3.06, 1.46–6.42), and pulmonary infarction (4; 17.07, 6.39–45.6). In monotherapy, both V and D showed unexpected clinically relevant SDRs for DIC (V: 3; 3.27, 1.05–10.16, D: 3; 8.37, 2.69–26.01). C was associated with ischaemic stroke (7; 33.98, 16.04–72.02), while T was associated with embolism (4; 17.76, 6.64–47.47) (Table S4).

## 4. Discussion

### 4.1. Descriptive Analysis

To the best of our knowledge, this study represents the largest and most comprehensive analysis of post-marketing AEs involving BRAF/MEKi for melanoma treatment, with a specific focus on CV toxicity, using data from a worldwide SRS database. Our findings indicate that a higher number of cases reported a BRAF/MEKi combination as the suspected drugs rather than a monotherapy. A higher overall number of possibly associated cases, including those reporting cAEs, could reflect the complexity of multi-drug interactions in line with evidence from clinical trials [4,9,11,12,19,25,26]. However, it should be noted that BRAF/MEKi combination therapies are the standard of care for BRAF V600-mutant metastatic melanoma, which accounts for approximately 40–50% of all melanoma cases [2,27]. Therefore, the higher number of associated reports may simply reflect their more frequent use compared to BRAFi monotherapies. A wide range of cAEs, occurring at different frequencies, have been reported in registration studies with BRAF/MEKi combinations in melanoma (see Supplementary Table S5). The TTO of cAEs varies significantly across studies, with cancer-therapy-related cardiac dysfunction potentially occurring anywhere from the first few weeks of therapy to up to two years after the completion of oncological treatment [28,29,30]. In our study, the median TTO of cAEs was shorter for combination therapies compared to monotherapies, suggesting that these AEs are more likely to manifest early, possibly due to additive cardiotoxic effects. The highest median value of TTO was observed for cases of E monotherapies. However, the skewed distribution of TTO for E in monotherapy appears to reflect a high variability in AE onset rather than a consistently delayed pattern. The wide Q1–Q3 observed in the boxplot suggests a dispersion of onset times, with some AEs occurring early and others substantially later. However, the extremely limited number of cases restricts the reliability of this observation. In such a small sample, even a few atypical reports can influence the distribution, making it difficult to discern whether the observed pattern reflects a true pharmacological characteristic or random variation. Although monotherapy theoretically allows for a clearer attribution of AEs to the drug, the interpretability of these results remains constrained by the low case count. Therefore, any conclusions must be drawn with caution.

This finding supports the hypothesis that BRAF/MEKi present a more immediate CV toxicity profile, emphasizing the need for the thorough baseline assessment and proactive monitoring of cAEs. In contrast, monotherapy may show a more delayed but sustained CV profile, requiring long-term surveillance. The considerable variability in onset patterns across different drug regimens underscores the importance of personalized mitigation strategies tailored to each treatment type and individual drug characteristics [17,18,31]. Available evidence suggests that MEKi are the primary drivers of CV toxicity, with BRAFi potentially enhancing their effects [30]. The underlying mechanisms are closely linked to the regulation of key molecules within the RAS/RAF/MEK/ERK pathway, which plays a crucial role in maintaining CV homeostasis [17,18,19]. Of note, the pathway has been shown to be involved in cardiomyocyte proliferation, survival and the adaption of the heart to both physiologic and pathologic conditions [18]. The blockade or deletion of cardiac ERK1/2 in mice promoted stress-induced apoptosis and heart failure [32]. Moreover, the RAS/RAF/MEK/ERK pathway plays a protective role in the vasculature through interaction with the vascular endothelial growth factor (VEGF) and other angiogenic factors, including the fibroblast growth factor (FGF) and platelet-derived growth factor (PDGF), and its inhibition can lead to an increased expression of CD47, which in turn inhibits nitric oxide stimulation with effects on vasculature tone and blood pressure [33]. Given the central role of this pathway in CV physiology, its inhibition is associated with a broad spectrum of cAEs [34]. However, it is important to underline that patients’ risk factors, including age and a personal history of pre-existing CV or even dysmetabolic comorbidities, could also play a significant role in predisposing them to the development of this peculiar toxicity. In light of this, current European Society of Cardiology (ESC) guidelines emphasize the importance of a thorough baseline risk stratification for patients undergoing BRAF/MEKi treatment. Recommended measures include baseline echocardiography for patients at a moderate to high risk of cardiotoxicity, blood pressure monitoring at each clinical visit, followed by weekly assessments during the first three months and monthly thereafter. Additionally, an ECG is advised at two and four weeks after initiating V + C therapy and every three months thereafter [30]. Given the increasing use of BRAF/MEKi in different cancer types, real-life safety data are crucial to identify known as well as novel toxicities occurring in patients outside clinical trials, which usually include highly selected populations without baseline significant comorbidities. These results could therefore be exploited by clinicians for implementing prevention, diagnostic, and therapeutic strategies.

### 4.2. Disproportionality Analysis

Several unexpected SDRs were detected for cAE-related PTs associated with the SMQs of interest, including cardiomyopathy, cardiac failure, arrhythmias, ischaemic heart disease, myocarditis, as well as embolic and thrombotic events. These findings have relevant implications for real-world clinical decision-making. In light of the potential CV SDRs detected, clinicians should consider a comprehensive baseline cardiac assessment prior to initiating BRAF/MEKi therapies, particularly in elderly patients or those with multiple comorbidities. Recommended evaluations may include electrocardiogram, echocardiography, and blood pressure monitoring, in accordance with current ESC cardio-oncology guidelines. Special consideration should be given to individuals with pre-existing CV disease, a history of exposure to cardiotoxic agents, or underlying pro-thrombotic conditions, who may benefit from closer surveillance or a multidisciplinary risk stratification approach. The distinct safety profiles observed across specific drug combinations may also support a more tailored therapeutic selection in high-risk populations. Although causality cannot be established from spontaneous reports, the robustness and clinical coherence of the signals observed reinforce the value of integrating pharmacovigilance insights into individualized treatment planning and long-term CV monitoring strategies [30].

#### 4.2.1. Cardiomyopathy

Considering cardiomyopathy, in a previous study, 27% of patients treated with BRAF/MEKi, represented by D + T in most cases, developed cardiac dysfunction. Cardiotoxicity was most frequently observed in patients classified as having a “low” or “medium” baseline cardiotoxicity risk (82%) [35]. In another retrospective study analyzing a large cohort of melanoma patients treated with D + T, 33% of patients experienced cardiotoxicity [36].

#### 4.2.2. Cardiac Failure

Regarding cardiac failure and left ventricular failure, a meta-analysis found that treatment with BRAF/MEKi (including D + T, V + C, and E + B) was associated with a decrease in the left ventricular ejection fraction (LVEF) compared to BRAFi monotherapy [37]. In a retrospective analysis, LVEF decrease was observed in 13.6% of melanoma patients treated with BRAF/MEKi combinations (in approximately 80% of cases) or BRAFi with, a median TTO of 11 months [38]. Interestingly, in another study, all clinically significant declines in LVEF with BRAF/MEKi occurred before the six-month assessment, with reversibility observed in 80% of cases [36]. A previous analysis based on the Truven Health Analytics/IBM MarketScan database reported an increased risk of heart failure with all combination therapies [39].

#### 4.2.3. Arrhythmias and Ischaemic Heart Disease

Another important SDR emerging from this study is the development of arrythmias. Considering bradyarrhythmia and monotherapy, a prolongation of the QT interval has been observed with V [25] but not with E [11], while contrasting results have been reported with D [4,40]. For tachyarrhythmias, D + T was associated with unexpected SDRs with clinical relevance for AF and ventricular arrhythmia. However, in a meta-analysis, the relative risks of AF and QTc prolongation were comparable for all combination therapies [37]. In a previous analysis, AF was reported in 2.7% of patients treated with BRAF/MEKi [39]. In an open-label multi-center safety study, AF was reported in 1.5% of patients treated with V [26]. For ischaemic heart disease, BRAFi treatment alone or in combination with a MEKi is associated with an increased risk of myocardial infarction; however, the relative risk was comparable [37].

#### 4.2.4. Myocarditis and Pericarditis

Considering myocarditis/pericarditis, specific data associated with these therapies are limited. This could be explained by the previous use of immune checkpoint inhibitors in melanoma treatment that may lead to life-threatening CV complications such as fulminant myocarditis and myopericarditis, which often result in the discontinuation of therapy [41,42].

#### 4.2.5. Embolic and Thrombotic Events

In our study, several unexpected SDRs with clinical relevance were identified for embolic and thrombotic events for all the three combinations: D + T was associated with important clinical manifestations, including DIC, splenic infarction and cerebral ischaemia; V + C was linked to cerebral ischaemia, pulmonary embolism and deep vein thrombosis; and E + B was associated with DIC and pulmonary infarction. Since no guidelines exist for screening or monitoring thromboembolism, these findings highlight the need for paying more attention to all associated clinical manifestations to enable early intervention and the treatment of these potentially fatal AEs. The development of thromboembolic events may be linked to cancer itself, as malignant cells can elevate the production and release of pro-coagulant factors, such as tissue factor. This leads to the activation of factor Xa, thereby amplifying the coagulation cascade. Additionally, tumor cells contribute to a pro-thrombotic environment by releasing pro-inflammatory cytokines and plasminogen activator inhibitor-1, which suppresses fibrinolysis and facilitates thrombus formation on vascular endothelial cells [43]. A pro-inflammatory state has been observed in patients treated with BRAF/MEKi [44]. BRAF/MEKi promote the upregulation and release of pro-coagulant factors, while simultaneously diminishing the activity of coagulation-inhibiting mechanisms. This disruption of hemostatic balance heightens the risk of thromboembolic events in patients undergoing these treatments [45]. Moreover, the use of BRAFi/MEKi has been associated with an increased risk of venous thromboembolism when compared with BRAFi monotherapy [19]. In a previous meta-analysis, the associated risk of pulmonary embolism was higher for melanoma patients treated with BRAF/MEKi (2.2%) compared with BRAFi monotherapy (0.4%) [37]. DIC is a severe and potentially life-threatening complication associated with melanoma, characterized by excessive activation of the coagulation system. Melanoma tumor cells exhibit pro-coagulant properties, contributing to coagulation dysregulation and subsequent fibrinolytic activation. Moreover, increased concentrations of thrombin–antithrombin and plasmin–antiplasmin complexes are linked to metastatic progression and a worse prognosis. Reports have documented cases of DIC following treatment with V, D, and T. In one instance, a patient developed DIC within five days of starting D + T but showed improvement with corticosteroid therapy. Another two cases of V-induced DIC, one of which led to a fatal intracerebral hemorrhage upon drug rechallenge, underscores the potential dangers of reintroducing BRAFi post-DIC. Despite the risks, targeted therapy with BRAF/MEKi has demonstrated efficacy in managing DIC in certain cases, delivering a rapid therapeutic response. However, careful monitoring is necessary due to the potential for exacerbating coagulopathy, highlighting the need for further research to optimize treatment strategies [46,47,48].

### 4.3. Strengths and Limitations

Our study has both strengths and limitations. SRS database analyses play a crucial role in pharmacovigilance due to their effectiveness in identifying SDRs that warrant further investigation. The FAERS database is particularly useful for detecting CV disorders linked to melanoma therapies, including unknown and serious cAEs [39]. Despite its extensive collection of reports, no prior research has specifically examined these SMQs in the context of melanoma. Thus, its primary strength lies in highlighting the CV toxicity associated with BRAF/MEKi therapies, thereby drawing increased attention from researchers and clinicians regarding this critical issue [49]. Given that such AEs may restrict the clinical use of highly effective targeted therapies, our findings emphasize the need for further investigation and early management strategies to mitigate CV toxicity in patients undergoing these treatments. It is now well established that patients with BRAF-mutant melanoma can benefit from both targeted therapies and immunotherapy. However, several critical questions remain regarding the optimal treatment selection and sequencing in this molecularly defined patient subgroup. Identifying patients at a higher risk of toxicity is essential for refining treatment strategies beyond molecular profiling [50]. Considerations should also include patient age, comorbidities—particularly preexisting CV conditions such as hypertension, prior heart failure/cardiomyopathy, and ischaemic disease—and other risk factors like diabetes mellitus and chronic kidney disease. Additionally, concomitant pharmacological therapies and potential drug–drug interactions should be carefully evaluated. In this context, it is worth noting that an increasing body of evidence supports the cardioprotective properties of sodium-glucose transport protein 2 (SGLT2) inhibitors across diverse clinical scenarios. These agents exert multiple beneficial effects, such as reducing myocardial inflammation and fibrosis, enhancing endothelial function, and improving cardiac energy metabolism, which may theoretically counteract the key pathophysiological mechanisms underlying BRAF/MEKi-induced cardiotoxicity. Notably, their capacity to preserve left ventricular function and limit adverse cardiac remodeling could be particularly relevant in attenuating the myocardial dysfunction associated with MAPK pathway inhibition [51]. Although specific evidence in the setting of BRAF V600–mutated melanoma is currently lacking, these agents may represent a potentially valuable adjunct for CV prevention. Therefore, in our view, further studies exploring this therapeutic hypothesis would be of considerable interest.

However, certain limitations related to SRS databases such as FAERS should be acknowledged when interpreting AE data [52,53]. The absence of a reliable denominator represents the first limitation. Thus, calculating the true incidence of the reported AEs is not feasible [54]. Furthermore, since AE reporting in the United States, although mandatory for pharmaceutical manufacturers, is voluntary for healthcare providers and patients, the possibility of underreporting cannot be excluded. Additionally, the completion of the reported information varies from case to case. This could have limited the number of cases available for analysis, thereby affecting our ability to assess certain aspects regarding the tolerability of these drugs, such as the TTO. Moreover, the FAERS database presents several intrinsic limitations that should be considered when interpreting the findings of this study. A major constraint is the lack of detailed clinical information—such as comprehensive medical history, longitudinal follow-up, and narrative case description—which hampers the assessment of baseline risk factors and the clinical context of AEs. Additionally, the database is structured around the AEs rather than patients, making it impossible to reliably link eventual subsequent cases to the same subject. This prevents accurate patient-level analysis and precludes the use of multivariable adjustment methods to control for potential confounders such as age, sex, comorbidities, or concomitant therapies. Moreover, missing or unstructured information, and the lack of temporal granularity further compound these limitations.

Another important concern is the possibility of duplicate reporting, a well-documented issue within FAERS that can lead to the overestimation of SDR strength and the misrepresentation of AE frequencies. To address this, we applied a rigorous multi-step deduplication process, as described in Section 2.

Furthermore, although disproportionality analysis remains a valuable tool for signal detection, it does not allow for causal inference. The lack of robust temporal associations between drug exposure and AE onset, combined with limited information on comorbidities and disease severity, restricts the ability to attribute events directly to specific drugs. Moreover, confounding by indication may be present, as advanced melanoma itself carries an inherent CV risk. The frequent use of polypharmacy and multiple lines of treatment in this patient population further complicates attribution. Lastly, potential channeling bias—where newer therapies may be preferentially prescribed to patients with more aggressive disease—cannot be ruled out [55]. These limitations highlight the need for cautious interpretation and suggest that complementary approaches, such as integration with structured clinical datasets or case-by-case evaluations, may be necessary to validate and refine these pharmacovigilance findings.

## 5. Conclusions

In conclusion, our findings suggest the potential value of comprehensive clinical assessment, both at baseline and during treatment, for the timely recognition and, where feasible, prevention of cAEs that may contribute to treatment discontinuation or more severe complications. While the identified SDRs related to arrhythmias, cardiac failure, ischaemic heart disease, and thrombo-embolic events warrant further investigation, they may also indicate a need to consider targeted preventive strategies in patients with elevated CV risk. Given the observational nature of pharmacovigilance data and its inherent limitations, such as underreporting, reporting bias, and a lack of detailed clinical information, these findings should be interpreted with caution and further complemented by prospective evaluations. Nevertheless, they reinforce the potential importance of integrated, multidisciplinary care. In particular, the involvement of a cardio-oncology team and structured collaboration between cardiologists and oncologists might contribute to more personalized risk stratification and management. Such an approach could support efforts to refine strategies for the early detection and mitigation of cardiotoxicity, aiming to optimize patient care in oncologic settings.

## Figures and Tables

**Table 1:** Characteristics of AE reports for BRAFi and MEKi in combination therapy and monotherapy.

Characteristic	Combination		Monotherapy	
	All AEs (*n* = 14,103)	cAEs (*n* = 1268)	All AEs (*n* = 4267)	cAEs (*n* = 323)
Age (years), median (Q1–Q3)	61 (51–70)	66 (55–74)	62 (51–71)	65 (58–74)
Age group, *n* (%)				
Neonate	1 (<0.1)		3 (0.1)	
Child	20 (0.1)	6 (0.5)	14 (0.3)	
Adolescent	7 (<0.1)	1 (0.1)	10 (0.2)	5 (1.5)
Adult	5493 (38.9)	469 (37)	1633 (38.3)	121 (37.5)
Elderly	3937 (27.9)	549 (43.3)	1233 (28.9)	146 (45.2)
Not available	4645 (32.9)	243 (19.2)	1374 (32.2)	51 (15.8)
Sex, *n* (%)				
Female	5624 (39.9)	519 (40.9)	1729 (40.5)	134 (41.5)
Male	6501 (46.1)	662 (52.2)	2266 (53.1)	171 (52.9)
Not available	1978 (14)	87 (6.9)	272 (6.4)	18 (5.6)
Weight (kg), median (Q1–Q3)	77 (65–90)	79 (66–90)	74 (62–87)	72 (59–86)
Type of reporter, *n* (%)				
Consumer	4370 (31)	235 (18.5)	1211 (28.4)	35 (10.8)
Physician	6144 (43.6)	692 (54.6)	1887 (44.2)	225 (69.7)
Pharmacist	689 (4.9)	38 (3)	360 (8.4)	10 (3.1)
Health-professional	1457 (10.3)	147 (11.6)	237 (5.6)	16 (5)
Other health-professional	1167 (8.3)	129 (10.2)	443 (10.4)	34 (10.5)
Not available	276 (2)	27 (2.1)	129 (3)	3 (0.9)
Outcome, *n* (%)				
Death	1717 (12.2)	114 (9)	615 (14.4)	33 (10.2)
Disability	152 (1.1)	22 (1.7)	57 (1.3)	3 (0.9)
Life-threatening	228 (1.6)	55 (4.3)	72 (1.7)	12 (3.7)
Hospitalization—initial or prolonged	4215 (29.9)	460 (36.3)	1078 (25.3)	149 (46.1)
Other serious (IME)	5251 (37.2)	602 (47.5)	1232 (28.9)	117 (36.2)
Required intervention to prevent permanent impairment/damage	2 (<0.1)		1 (<0.1)	1 (0.3)
Not available	2538 (18)	15 (1.2)	1212 (28.4)	8 (2.5)
Reporter country, *n* (%)				
Africa	28 (0.2)		25 (0.6)	
Asia	1595 (11.3)	159 (12.5)	309 (7.2)	42 (13)
Europe	5596 (39.7)	762 (60.1)	1323 (31)	175 (54.2)
North America	5102 (36.2)	206 (16.2)	2337 (54.8)	85 (26.3)
Central America	17 (0.1)	2 (0.2)	17 (0.4)	3 (0.9)
South America	420 (3)	39 (3.1)	60 (1.4)	6 (1.9)
Oceania	200 (1.4)	23 (1.8)	76 (1.8)	11 (3.4)
Not available	1145 (8.1)	77 (6.1)	120 (2.8)	1 (0.3)
Year of reporting, *n* (%)				
2014	207 (1.5)	21 (1.7)	394 (9.2)	35 (10.8)
2015	1055 (7.5)	72 (5.7)	904 (21.2)	78 (24.1)
2016	1130 (8)	96 (7.6)	975 (22.8)	55 (17)
2017	1226 (8.7)	126 (9.9)	352 (8.2)	34 (10.5)
2018	1786 (12.7)	159 (12.5)	340 (8)	35 (10.8)
2019	2430 (17.2)	209 (16.5)	312 (7.3)	35 (10.8)
2020	1887 (13.4)	190 (15)	350 (8.2)	19 (5.9)
2021	1714 (12.2)	132 (10.4)	230 (5.4)	14 (4.3)
2022	1317 (9.3)	150 (11.8)	236 (5.5)	9 (2.8)
2023	1351 (9.6)	113 (8.9)	174 (4.1)	9 (2.8)

Abbreviations: AE, adverse event; BRAFi, BRAF inhibitor; cAE, cardiovascular adverse event; IME, Important Medical Event; Kg, kilograms; MEKi, MEK inhibitor; Q1, quartile 1; Q3, quartile 3.

**Table 2:** Disproportionality analysis for SMQs of interest with BRAFi and MEKi in combination therapy.

	D + T			V + C			E + B		
	N	ROR (95% CI)	Expected	N	ROR (95% CI)	Expected	N	ROR (95% CI)	Expected
**Bradyarrhythmias**									
Atrioventricular block complete	6	3.24 (1.45–7.22)	Yes						
Brugada syndrome	4	25.12 (9.35–67.45)	No						
Electrocardiogram QRS complex prolonged	5	6.53 (2.71–15.73)	No						
Electrocardiogram QT prolonged	59	5.09 (3.94–6.58)	No	26	9.5 (6.45–13.98)	Yes	14	3.46 (2.04–5.84)	Yes
Electrocardiogram repolarisation abnormality	8	32.89 (16.33–66.27)	No						
Atrioventricular block *	4	-							
Atrioventricular block first degree *	5	3.98 (1.66–9.58)	Yes						
Atrioventricular block second degree *	3	3.52 (1.13–10.92)	Yes						
Bundle branch block left *	5	4.14 (1.72–9.97)	Yes						
Bundle branch block right *	5	3.97 (1.65–9.56)	Yes	5	16.78 (6.97–40.4)	No			
Defect conduction intraventricular *	6	49.2 (21.83–110.91)	No				3	69.62 (22.25–217.87)	No
Sinus arrhythmia *	3	7.37 (2.37–22.92)	No						
Sinus bradycardia *	10	3.64 (1.95–6.76)	Yes				6	6.27 (2.81–13.97)	No
**Cardiac failure**									
Cardiac failure	67	2.05 (1.61–2.6)	Yes	29	3.76 (2.6–5.42)	No	18	-	
Cardiac failure chronic	3	-							
Cardiac failure congestive	9	-							
Cardiogenic shock							5	3.91 (1.63–9.4)	No
Left ventricular failure				10	32.56 (17.47–60.7)	No	4	8.82 (3.31–23.54)	Yes
Pulmonary oedema	15	-							
**Cardiomyopathy**									
Cardiomyopathy	16	3.7 (2.26–6.04)	Yes	4	3.89 (1.46–10.37)	Yes	3	-	
Cardiotoxicity	7	4.22 (2.01–8.87)	No						
Dilated cardiomyopathy	12	7.17 (4.07–12.65)	Yes						
Ischaemic cardiomyopathy	6	6.44 (2.89–14.37)	Yes						
Tachycardia induced cardiomyopathy *	7	135.12 (62.43–292.43)	Yes						
Ejection fraction abnormal *	4	5.95 (2.23–15.9)	Yes						
Ejection fraction decreased *	148	24.58 (20.87–28.95)	Yes	36	24.93 (17.92–34.67)	Yes	17	7.91 (4.91–12.75)	Yes
Stress cardiomyopathy *				5	16.61 (6.9–39.98)	Yes			
**Tachyarrhythmias**									
Atrial fibrillation	99	2.37 (1.94–2.89)	No	27	2.73 (1.86–3.99)	Yes	8	-	
Ventricular arrhythmia	4	4.13 (1.55–11.01)	No	5	21.8 (9.05–52.49)	Yes			
Atrial flutter *	5	-					3	-	
Extrasystoles *	3	-		4	6.39 (2.39–17.05)	No			
Sinus tachycardia *	8	2.04 (1.02–4.08)	No						
Supraventricular tachycardia *	5								
Tachyarrhythmia *	8	9.07 (4.53–18.18)	No						
Ventricular extrasystoles *	3	-		4	5.44 (2.04–14.52)	No			
**Ischaemic heart disease**									
Acute myocardial infarction	8	-		5	-		3	-	
Myocardial infarction	30	-		5	-		8	-	
Angina pectoris	14	-					7	-	
Coronary artery stenosis	10	7.28 (3.91–13.55)	No						
Myocardial ischaemia	3	-							
Coronary artery disease *	6	-							
Blood creatine phosphokinase MB increased *	7	25.95 (12.29–54.77)	No						
Troponin T increased *	11	24.36 (13.43–44.2)	No						
Troponin increased *	6	-							
**Embolism and thrombotic events**									
Disseminated intravascular coagulation	38	10.22 (7.42–14.06)	No				4	3.06 (1.15–8.17)	No
Splenic infarction	9	15.78 (8.18–30.43)	No						
Blindness transient							5	4.78 (1.99–11.49)	No
Retinal vein occlusion	14	14.93 (8.82–25.29)	Yes	4	17.87 (6.69–47.72)	Yes	3	9.12 (2.94–28.33)	Yes
Cerebral infarction	11	-					3	-	
Cerebral ischaemia	12	7.24 (4.11–12.77)	No	8	20.37 (10.16–40.83)	No			
Cerebrovascular accident	77	-		11	-		15	-	
Haemorrhagic stroke	6	-							
Hemiparesis	29	4.42 (3.07–6.37)	No				7	3.06 (1.46–6.42)	No
Hemiplegia	10	3.06 (1.64–5.68)	No						
Ischaemic stroke	6	-							
Monoplegia	4	-							
Paraparesis	7	11.1 (5.28–23.35)	No						
Paraplegia	9	8.47 (4.4–16.3)	No						
Quadriplegia	3	4.84 (1.56–15.04)	No						
Transient ischaemic attack	7	-					6	-	
Pulmonary embolism	111	3.35 (2.78–4.04)	Yes	22	2.79 (1.83–4.24)	No	16	-	
Pulmonary infarction							4	17.07 (6.39–45.6)	No
Pulmonary thrombosis	6	-							
Deep vein thrombosis	35	1.59 (1.14–2.21)	Yes	10	1.91 (1.03–3.55)	No	4	-	
Embolism	4	-					9	8.95 (4.65–17.23)	Yes
Subclavian vein thrombosis	3	7.88 (2.54–24.52)	Yes						
Thrombosis	52	1.47 (1.12–1.93)	Yes	4	-		10	-	
Monoparesis *				8	43.49 (21.67–87.28)	No			
Paradoxical embolism *	7	230.83 (104.42–510.27)	No						
Superficial vein thrombosis *	3	-							
Thrombophlebitis *	7	5.47 (2.6–11.49)	Yes						
Venous thrombosis *	10	7.28 (3.91–13.55)	Yes						
Venous thrombosis limb *	4	4.18 (1.57–11.15)	Yes						

Abbreviations: B, binimetinib; BRAFi, BRAF inhibitor; C, cobimetinib; CI, confidence interval; D, dabrafenib; E, encorafenib; MEKi, MEK inhibitor; ROR, reporting odd ratio; SMQ, Standardized MedDRA Query; T, trametinib; V, vemurafenib. * Preferred Term not included in the Important Medical Event (IME) list.

## Data Availability

This study was entirely based on publicly anonymized data made available by the Food and Drug Administration. The raw data can be downloaded at the following link: https://fis.fda.gov/extensions/FPD-QDE-FAERS/FPD-QDE-FAERS.html (accessed on 29 January 2024).

## Supplementary Materials

The following supporting information can be downloaded at: https://www.mdpi.com/article/10.3390/cancers17111755/s1, Table S1: List of Preferred Term considered for each Standardized MedDRA Query of interest; Table S2: Characteristics of AE reports for BRAFi and MEKi as combination therapy; Table S3: Characteristics of AE reports for BRAFi and MEKi as monotherapy; Table S4: Disproportionality analysis for SMQs of interest with BRAFi and MEKi in monotherapy; Table S5: Selected cardiovascular adverse events reported in main registration studies.
