# Supplementary material for: Cardiovascular Safety Profile of BRAF and MEK Inhibitors in Melanoma: FAERS Data Through a Retrospective Disproportionality Analysis (2014–2023)
Source: Cancers (Basel). 2025 May 23;17(11):1755. doi: 10.3390/cancers17111755 (PMC12153699; doi:10.3390/cancers17111755)
Supplement: Supplementary file 1 [file cancers-17-01755-s001.zip › cancers-3560515-supplementary.pdf]

Table S1. List of Preferred Term considered for each Standardized MedDRA Query of interest

**Bradyarrhythmia**
Bradyarrhythmia
Ictal bradycardia syndrome
Ventricular asystole
Accessory cardiac pathway
Adams-Stokes syndrome
Agonal rhythm
Atrial conduction time prolongation
Atrial escape rhythm
Atrial standstill
Atrioventricular block
Atrioventricular block complete
Atrioventricular block first degree
Atrioventricular block second degree
Atrioventricular conduction time shortened
Atrioventricular dissociation
Atrioventricular node dysfunction
Bifascicular block
BRASH syndrome
Brugada syndrome
Bundle branch block
Bundle branch block bilateral
Bundle branch block left
Bundle branch block right
Conduction disorder
Defect conduction intraventricular
Ectopic atrial rhythm
Electrocardiogram delta waves abnormal
Electrocardiogram PR prolongation
Electrocardiogram PR shortened
Electrocardiogram QRS complex prolonged
Electrocardiogram QT prolonged
Electrocardiogram repolarisation abnormality
Fascicular block
Lenegre's disease
Long QT syndrome
Paroxysmal atrioventricular block
Sinoatrial block
Trifascicular block
Ventricular dyssynchrony

Wolff-Parkinson-White syndrome
Atrial escape rhythm
Ectopic atrial rhythm
Nodal arrhythmia
Nodal rhythm
Sinus arrest
Sinus arrhythmia
Sinus bradycardia
Sinus node dysfunction
Wandering pacemaker
**Tachyarrhythmias**
Arrhythmia supraventricular
Atrial fibrillation
Atrial flutter
Atrial parasystole
Atrial tachycardia
Congenital supraventricular tachycardia
Familial atrial fibrillation
Frederick's syndrome
Junctional ectopic tachycardia
Sinus tachycardia
Supraventricular extrasystoles
Supraventricular tachyarrhythmia
Supraventricular tachycardia
Anomalous atrioventricular excitation
Cardiac fibrillation
Cardiac flutter
Extrasystoles
Tachyarrhythmia
Accelerated idioventricular rhythm
Arrhythmic storm
Cardiac fibrillation
Early repolarisation syndrome
Parasystole
Rhythm idioventricular
Torsade de pointes
Ventricular arrhythmia
Ventricular extrasystoles
Ventricular fibrillation
Ventricular flutter
Ventricular parasystole
Ventricular pre-excitation

Ventricular tachyarrhythmia
Ventricular tachycardia
**Noninfectious myocarditis/pericarditis**
Autoimmune myocarditis
Autoimmune pericarditis
Carditis
Chronic myocarditis
Eosinophilic myocarditis
Giant cell myocarditis
Hypersensitivity myocarditis
Immune-mediated myocarditis
Immune-mediated pericarditis
Myocarditis
Myopericarditis
Pericarditis
Pericarditis adhesive
Pericarditis constrictive
Pleuropericarditis
**Myocardial infarction**
Acute cardiac event
Acute coronary syndrome
Acute myocardial infarction
Angina unstable
Blood creatine phosphokinase MB abnormal
Blood creatine phosphokinase MB increased
Coronary artery embolism
Coronary artery occlusion
Coronary artery reocclusion
Coronary artery thrombosis
Coronary bypass thrombosis
Coronary vascular graft occlusion
Heart-type fatty acid-binding protein increased
Kounis syndrome
Myocardial infarction
Myocardial necrosis
Myocardial reperfusion injury
Myocardial stunning
Papillary muscle infarction
Periprocedural myocardial infarction
Post procedural myocardial infarction
Postinfarction angina
Silent myocardial infarction

Troponin I increased
Troponin increased
Troponin T increased
**Cardiac failure**
Acute left ventricular failure
Acute pulmonary oedema
Acute right ventricular failure
Cardiac asthma
Cardiac failure
Cardiac failure acute
Cardiac failure chronic
Cardiac failure congestive
Cardiac failure high output
Cardiogenic shock
Cardiohepatic syndrome
Cardiopulmonary failure
Cardiorenal syndrome
Chronic left ventricular failure
Chronic right ventricular failure
Congestive hepatopathy
Cor pulmonale
Cor pulmonale acute
Cor pulmonale chronic
Ejection fraction decreased
Hepatojugular reflux
Left ventricular failure
Low cardiac output syndrome
Neonatal cardiac failure
Obstructive shock
Pulmonary oedema
Pulmonary oedema neonatal
Radiation associated cardiac failure
Right ventricular ejection fraction decreased
Right ventricular failure
Ventricular failure
**Cardiomyopathy**
Atrial septal defect acquired
Biopsy heart abnormal
Cardiac amyloidosis
Cardiac hypertrophy
Cardiac iron overload
Cardiac sarcoidosis

Cardiac septal hypertrophy
Cardiomyopathy
Cardiomyopathy acute
Cardiomyopathy alcoholic
Cardiomyopathy neonatal
Cardiotoxicity
Chagas' cardiomyopathy
Diabetic cardiomyopathy
Dilated cardiomyopathy
Ejection fraction abnormal
Ejection fraction decreased
Eosinophilic myocarditis
Giant cell myocarditis
HIV cardiomyopathy
Hypertensive cardiomyopathy
Hypertrophic cardiomyopathy
Ischaemic cardiomyopathy
Metabolic cardiomyopathy
Mitochondrial cardiomyopathy
Myocardial calcification
Myocardial fibrosis
Myocardial haemorrhage
Non-obstructive cardiomyopathy
Obesity cardiomyopathy
Pacing induced cardiomyopathy
Peripartum cardiomyopathy
Pulmonary arterial wedge pressure increased
Restrictive cardiomyopathy
Right ventricular ejection fraction decreased
Septic cardiomyopathy
Stress cardiomyopathy
Tachycardia induced cardiomyopathy
Thyrotoxic cardiomyopathy
Toxic cardiomyopathy
Uraemic cardiomyopathy
Ventricular septal defect acquired
Viral cardiomyopathy
**Embolic and thrombotic events**
Acute aortic syndrome
Acute coronary syndrome
Acute myocardial infarction
Amaurosis

Amaurosis fugax
Aneurysm thrombosis
Angioplasty
Angiostomy
Aortic aneurysm thrombosis
Aortic bypass
Aortic embolus
Aortic surgery
Aortic thrombosis
Aortogram abnormal
Arterectomy
Arterectomy with graft replacement
Arterial angioplasty
Arterial bypass operation
Arterial graft
Arterial occlusive disease
Arterial recanalisation procedure
Arterial revascularisation
Arterial stent insertion
Arterial therapeutic procedure
Arterial thrombosis
Arteriogram abnormal
Arteriogram carotid abnormal
Arteriotomy
Atherectomy
Atherosclerotic plaque rupture
Atrial appendage closure
Atrial appendage resection
Basal ganglia infarction
Basilar artery occlusion
Basilar artery thrombosis
Blindness transient
Brachiocephalic artery occlusion
Capsular warning syndrome
Carotid angioplasty
Carotid arterial embolus
Carotid artery bypass
Carotid artery occlusion
Carotid artery stent insertion
Carotid artery thrombosis
Carotid endarterectomy
Carotid revascularisation

Cerebellar artery occlusion
Cerebellar artery thrombosis
Cerebral angioplasty
Cerebral artery embolism
Cerebral artery occlusion
Cerebral artery stent insertion
Cerebral artery thrombosis
Cerebral bypass surgery
Cerebral hypoperfusion
Cerebral revascularisation
Cerebrovascular insufficiency
Cerebrovascular stenosis
Coeliac artery occlusion
Coronary angioplasty
Coronary arterial stent insertion
Coronary artery bypass
Coronary artery embolism
Coronary artery occlusion
Coronary artery reocclusion
Coronary artery surgery
Coronary artery thrombosis
Coronary endarterectomy
Coronary revascularisation
Coronary vascular graft occlusion
Embolia cutis medicamentosa
Embolism arterial
Endarterectomy
Femoral artery embolism
Hepatic artery embolism
Hepatic artery occlusion
Hepatic artery thrombosis
Hypothenar hammer syndrome
Iliac artery embolism
Iliac artery occlusion
Incomplete atrial appendage closure
Internal capsule infarction
Intra-aortic balloon placement
Intraoperative cerebral artery occlusion
Ischaemic cerebral infarction
Ischaemic stroke
Lacunar infarction
Left atrial appendage closure implant

Leriche syndrome
Mesenteric arterial occlusion
Mesenteric arteriosclerosis
Mesenteric artery embolism
Mesenteric artery stenosis
Mesenteric artery stent insertion
Mesenteric artery thrombosis
Metabolic stroke
Myocardial infarction
Myocardial necrosis
Ophthalmic artery occlusion
Ophthalmic artery thrombosis
Papillary muscle infarction
Penile artery occlusion
Percutaneous coronary intervention
Peripheral arterial occlusive disease
Peripheral arterial reocclusion
Peripheral artery angioplasty
Peripheral artery bypass
Peripheral artery occlusion
Peripheral artery stent insertion
Peripheral artery surgery
Peripheral artery thrombosis
Peripheral embolism
Peripheral endarterectomy
Popliteal artery entrapment syndrome
Post procedural myocardial infarction
Postinfarction angina
Precerebral artery embolism
Precerebral artery occlusion
Precerebral artery thrombosis
Profundaplasty
Pseudo-occlusion of internal carotid artery
Pulmonary angioplasty
Pulmonary artery occlusion
Pulmonary artery stent insertion
Pulmonary artery therapeutic procedure
Pulmonary artery thrombosis
Pulmonary endarterectomy
Pulmonary tumour thrombotic microangiopathy
Renal artery angioplasty
Renal artery occlusion

Renal artery revascularisation
Renal artery thrombosis
Renal embolism
Renal-limited thrombotic microangiopathy
Retinal artery embolism
Retinal artery occlusion
Retinal artery thrombosis
Segmental arterial mediolysis
Silent myocardial infarction
Spinal artery embolism
Spinal artery thrombosis
Splenic artery thrombosis
Splenic embolism
Stress cardiomyopathy
Subclavian artery embolism
Subclavian artery occlusion
Subclavian artery thrombosis
Thromboembolectomy
Thrombotic microangiopathy
Thrombotic thrombocytopenic purpura
Transient ischaemic attack
Truncus coeliacus thrombosis
Vascular pseudoaneurysm thrombosis
Vertebral artery occlusion
Vertebral artery thrombosis
Vertebrobasilar infarction
Visual acuity reduced transiently
Aseptic cavernous sinus thrombosis
Axillary vein thrombosis
Brachiocephalic vein occlusion
Brachiocephalic vein thrombosis
Budd-Chiari syndrome
Catheterisation venous
Cavernous sinus thrombosis
Central venous catheterisation
Cerebral venous sinus thrombosis
Cerebral venous thrombosis
Compression garment application
Deep vein thrombosis
Deep vein thrombosis postoperative
Embolism venous
Hepatic vein embolism

Hepatic vein occlusion
Hepatic vein thrombosis
Homans' sign positive
Iliac vein occlusion
Inferior vena cava syndrome
Inferior vena caval occlusion
Jugular vein embolism
Jugular vein occlusion
Jugular vein thrombosis
Mahler sign
May-Thurner syndrome
Mesenteric vein embolism
Mesenteric vein thrombosis
Mesenteric venous occlusion
Obstetrical pulmonary embolism
Obstructive shock
Ophthalmic vein thrombosis
Ovarian vein thrombosis
Paget-Schroetter syndrome
Pelvic venous thrombosis
Penile vein thrombosis
Peripheral vein occlusion
Peripheral vein thrombosis
Peripheral vein thrombus extension
Phlebectomy
Portal vein cavernous transformation
Portal vein embolism
Portal vein occlusion
Portal vein thrombosis
Portosplenomesenteric venous thrombosis
Post procedural pulmonary embolism
Post thrombotic syndrome
Postoperative thrombosis
Postpartum venous thrombosis
Pulmonary embolism
Pulmonary infarction
Pulmonary microemboli
Pulmonary thrombosis
Pulmonary vein occlusion
Pulmonary veno-occlusive disease
Pulmonary venous thrombosis
Renal vein embolism

Renal vein occlusion
Renal vein thrombosis
Retinal vein occlusion
Retinal vein thrombosis
SI QIII TIII pattern
Sigmoid sinus thrombosis
Spermatic vein thrombosis
Splenic vein occlusion
Splenic vein thrombosis
Subclavian vein embolism
Subclavian vein occlusion
Subclavian vein thrombosis
Superficial vein thrombosis
Superior sagittal sinus thrombosis
Superior vena cava occlusion
Superior vena cava syndrome
Thrombophlebitis
Thrombophlebitis migrans
Thrombophlebitis neonatal
Thrombosed varicose vein
Thrombosis corpora cavernosa
Transverse sinus thrombosis
Vena cava embolism
Vena cava filter insertion
Vena cava filter removal
Vena cava thrombosis
Venogram abnormal
Venoocclusive disease
Venoocclusive liver disease
Venous angioplasty
Venous occlusion
Venous operation
Venous recanalisation
Venous repair
Venous stent insertion
Venous thrombosis
Venous thrombosis in pregnancy
Venous thrombosis limb
Venous thrombosis neonatal
Visceral venous thrombosis
Administration site thrombosis
Adrenal thrombosis

Angiogram abnormal
Angiogram cerebral abnormal
Angiogram peripheral abnormal
Antiphospholipid syndrome
Application site thrombosis
Arteriovenous fistula occlusion
Arteriovenous fistula thrombosis
Arteriovenous graft thrombosis
Artificial blood vessel occlusion
Atrial thrombosis
Autoimmune heparin-induced thrombocytopenia
Basal ganglia stroke
Bone infarction
Brain stem embolism
Brain stem infarction
Brain stem stroke
Brain stem thrombosis
Cardiac ventricular thrombosis
Catheter directed thrombolysis
Catheter site thrombosis
Cerebellar embolism
Cerebellar infarction
Cerebral congestion
Cerebral infarction
Cerebral infarction foetal
Cerebral ischaemia
Cerebral microembolism
Cerebral microinfarction
Cerebral septic infarct
Cerebral thrombosis
Cerebral vascular occlusion
Cerebrospinal thrombotic tamponade
Cerebrovascular accident
Cerebrovascular accident prophylaxis
Cerebrovascular disorder
Cerebrovascular operation
Choroidal infarction
Collateral circulation
Coronary bypass thrombosis
Device embolisation
Device occlusion
Device related thrombosis

Diplegia
Directional Doppler flow tests abnormal
Disseminated intravascular coagulation
Disseminated intravascular coagulation in newborn
Embolic cerebellar infarction
Embolic cerebral infarction
Embolic pneumonia
Embolic stroke
Embolism
Eye infarction
Fluorescence angiogram abnormal
Foetal cerebrovascular disorder
Foetal vascular malperfusion
Gastric infarction
Graft thrombosis
Haemorrhagic adrenal infarction
Haemorrhagic cerebral infarction
Haemorrhagic infarction
Haemorrhagic stroke
Haemorrhagic transformation stroke
Haemorrhoids thrombosed
Hemiparesis
Hemiplegia
Heparin-induced thrombocytopenia
Hepatic infarction
Hepatic vascular thrombosis
Implant site thrombosis
Incision site vessel occlusion
Infarction
Infusion site thrombosis
Injection site thrombosis
Inner ear infarction
Instillation site thrombosis
Intestinal infarction
Intracardiac mass
Intracardiac thrombus
Lambl's excrescences
Medical device site thrombosis
Mesenteric vascular insufficiency
Mesenteric vascular occlusion
Microembolism
Middle cerebral artery stroke

Monoparesis
Monoplegia
Muscle infarction
Occipital lobe stroke
Ophthalmic vascular thrombosis
Optic nerve infarction
Pancreatic infarction
Paradoxical embolism
Paraneoplastic thrombosis
Paraparesis
Paraplegia
Paresis
Parietal lobe stroke
Peripheral revascularisation
Pituitary infarction
Placental infarction
Pneumatic compression therapy
Portal shunt procedure
Post procedural stroke
Postpartum thrombosis
Prosthetic cardiac valve thrombosis
Prosthetic vessel implantation
Quadriparesis
Quadriplegia
Renal infarct
Renal vascular thrombosis
Retinal infarction
Retinal vascular thrombosis
Revascularisation procedure
Shunt occlusion
Shunt thrombosis
Spinal cord infarction
Spinal stroke
Splenic infarction
Splenic thrombosis
Spontaneous heparin-induced thrombocytopenia syndrome
Stoma site thrombosis
Stroke in evolution
Strokectomy
Surgical vascular shunt
Testicular infarction
Thalamic infarction

Thalamic stroke
Thrombectomy
Thromboangiitis obliterans
Thrombolysis
Thrombosis
Thrombosis in device
Thrombosis mesenteric vessel
Thrombosis prophylaxis
Thrombosis with thrombocytopenia syndrome
Thrombotic cerebral infarction
Thrombotic stroke
Thyroid infarction
Tricuspid valve thrombosis
Tumour embolism
Tumour thrombectomy
Tumour thrombosis
Ultrasonic angiogram abnormal
Ultrasound Doppler abnormal
Umbilical cord occlusion
Umbilical cord thrombosis
Vaccination site thrombosis
Vascular access site thrombosis
Vascular device occlusion
Vascular graft
Vascular graft occlusion
Vascular graft thrombosis
Vascular operation
Vascular stent insertion
Vascular stent occlusion
Vascular stent thrombosis
Vasodilation procedure
Vessel puncture site occlusion
Vessel puncture site thrombosis
Visual midline shift syndrome
**Ischaemic heart disease**
Acute cardiac event
Acute coronary syndrome
Acute myocardial infarction
Angina unstable
Blood creatine phosphokinase MB abnormal
Blood creatine phosphokinase MB increased
Coronary artery embolism

Coronary artery occlusion
Coronary artery reocclusion
Coronary artery thrombosis
Coronary bypass thrombosis
Coronary vascular graft occlusion
Heart-type fatty acid-binding protein increased
Kounis syndrome
Myocardial infarction
Myocardial necrosis
Myocardial reperfusion injury
Myocardial stunning
Papillary muscle infarction
Periprocedural myocardial infarction
Post procedural myocardial infarction
Postinfarction angina
Silent myocardial infarction
Troponin I increased
Troponin increased
Troponin T increased
Acute cardiac event
Angina pectoris
Angina unstable
Anginal equivalent
Arteriosclerosis coronary artery
Arteriospasm coronary
Cardiac perfusion defect
Chronic coronary syndrome
Coronary angioplasty
Coronary arterial stent insertion
Coronary artery bypass
Coronary artery compression
Coronary artery disease
Coronary artery dissection
Coronary artery insufficiency
Coronary artery restenosis
Coronary artery stenosis
Coronary artery surgery
Coronary brachytherapy
Coronary bypass stenosis
Coronary endarterectomy
Coronary no-reflow phenomenon
Coronary ostial stenosis

Coronary revascularisation
Coronary slow flow phenomenon
Coronary steal syndrome
Coronary vascular graft stenosis
Diabetic coronary microangiopathy
ECG signs of myocardial ischaemia
External counterpulsation
Haemorrhage coronary artery
Ischaemic cardiomyopathy
Ischaemic mitral regurgitation
Microvascular coronary artery disease
Myocardial hypoperfusion
Myocardial hypoxia
Myocardial ischaemia
Percutaneous coronary intervention
Prinzmetal angina
Stress cardiomyopathy
Subclavian coronary steal syndrome
Subendocardial ischaemia
Wellens' syndrome

Table S2. Characteristics of AE reports for BRAFi and MEKi as combination therapy.

Characteristic	D+T		E+B		V+C		Combination	
	All AEs (*n* = 8895)	cAEs (*n* = 890)	All AEs (*n* = 3097)	cAEs (*n* = 170)	All AEs (*n* = 2111)	cAEs (*n* = 208)	All AEs (*n* = 14,103)	cAEs (*n* = 1268)
Age (years), median (Q1-Q3)	60 (49 - 70)	64 (54 - 74)	65 (55 - 73)	69 (60 - 77)	60 (50 - 70)	69 (55 - 76)	61 (51 – 70)	66 (55 - 74)
Age group, *n* (%)								
Neonate	1 (<0.1)						1 (<0.1)	
Child	19 (0.2)	6 (0.7)	1 (<0.1)				20 (0.1)	6 (0.5)
Adolescent	6 (0.1)	1 (0.1)	1 (<0.1)				7 (<0.1)	1 (0.1)
Adult	3550 (39.9)	347 (39)	887 (28.6)	46 (27.1)	1056 (50)	76 (36.5)	5493 (38.9)	469 (37)
Elderly	2260 (25.4)	351 (39.4)	940 (30.4)	79 (46.5)	737 (34.9)	119 (57.2)	3937 (27.9)	549 (43.3)
Not available	3059 (34.4)	185 (20.8)	1268 (40.9)	45 (26.5)	318 (15.1)	13 (6.2)	4645 (32.9)	243 (19.2)
Sex, *n* (%)								
Female	3768 (42.4)	370 (41.6)	894 (28.9)	51 (30)	962 (45.6)	98 (47.1)	5624 (39.9)	519 (40.9)
Male	4364 (49.1)	477 (53.6)	1050 (33.9)	80 (47.1)	1087 (51.5)	105 (50.5)	6501 (46.1)	662 (52.2)
Not available	763 (8.6)	43 (4.8)	1153 (37.2)	39 (22.9)	62 (2.9)	5 (2.4)	1978 (14)	87 (6.9)
Weight (Kg), median (Q1-Q3)	77 (65 - 90)	80 (68 - 90)	75 (63 - 88)	76 (66 - 92)	77 (65 - 89)	80 (65 - 90)	77 (65 – 90)	79 (66 - 90)
Type of reporter, *n* (%)								
Consumer	2684 (30.2)	184 (20.7)	1471 (47.5)	40 (23.5)	215 (10.2)	11 (5.3)	4370 (31)	235 (18.5)
Physician	3646 (41)	461 (51.8)	829 (26.8)	55 (32.4)	1669 (79.1)	176 (84.6)	6144 (43.6)	692 (54.6)
Pharmacist	466 (5.2)	28 (3.1)	136 (4.4)	4 (2.4)	87 (4.1)	6 (2.9)	689 (4.9)	38 (3)
Health-professional	864 (9.7)	73 (8.2)	557 (18)	69 (40.6)	36 (1.7)	5 (2.4)	1457 (10.3)	147 (11.6)
Other health-professional	1055 (11.9)	119 (13.4)	19 (0.6)		93 (4.4)	10 (4.8)	1167 (8.3)	129 (10.2)
Not available	180 (2)	25 (2.8)	85 (2.7)	2 (1.2)	11 (0.5)		276 (2)	27 (2.1)
Outcome, *n* (%)								
Death	1269 (14.3)	87 (9.8)	268 (8.7)	13 (7.6)	180 (8.5)	14 (6.7)	1717 (12.2)	114 (9)
Disability	97 (1.1)	13 (1.5)	16 (0.5)	4 (2.4)	39 (1.8)	5 (2.4)	152 (1.1)	22 (1.7)
Life-threatening	159 (1.8)	40 (4.5)	24 (0.8)	5 (2.9)	45 (2.1)	10 (4.8)	228 (1.6)	55 (4.3)
Hospitalization - initial or prolonged	2459 (27.6)	305 (34.3)	693 (22.4)	53 (31.2)	1063 (50.4)	102 (49)	4215 (29.9)	460 (36.3)
Other serious (IME)	3488 (39.2)	439 (49.3)	1074 (34.7)	89 (52.4)	689 (32.6)	74 (35.6)	5251 (37.2)	602 (47.5)
Required intervention to prevent permanent impairment/damage	2 (<0.1)						2 (<0.1)	
Not available	1421 (16)	6 (0.7)	1022 (33)	6 (3.5)	95 (4.5)	3 (1.4)	2538 (18)	15 (1.2)
Reporter country, *n* (%)								
Africa	25 (0.3)				3 (0.1)		28 (0.2)	
Asia	996 (11.2)	141 (15.8)	419 (13.5)	11 (6.5)	180 (8.5)	7 (3.4)	1595 (11.3)	159 (12.5)
Europe	3281 (36.9)	481 (54)	874 (28.2)	108 (63.5)	1441 (68.3)	173 (83.2)	5596 (39.7)	762 (60.1)
North America	2942 (33.1)	133 (14.9)	1776 (57.3)	49 (28.8)	384 (18.2)	24 (11.5)	5102 (36.2)	206 (16.2)
Central America	14 (0.2)	2 (0.2)			3 (0.1)		17 (0.1)	2 (0.2)
South America	348 (3.9)	35 (3.9)	10 (0.3)		62 (2.9)	4 (1.9)	420 (3)	39 (3.1)
Oceania	151 (1.7)	21 (2.4)	12 (0.4)	2 (1.2)	37 (1.8)		200 (1.4)	23 (1.8)

Not available	1138 (12.8)	77 (8.7)	6 (0.2)		1 (<0.1)		1145 (8.1)	77 (6.1)
Year of reporting, *n* (%)								
2014	195 (2.2)	20 (2.2)			12 (0.6)	1 (0.5)	207 (1.5)	21 (1.7)
2015	987 (11.1)	65 (7.3)			68 (3.2)	7 (3.4)	1055 (7.5)	72 (5.7)
2016	822 (9.2)	71 (8)			308 (14.6)	25 (12)	1130 (8)	96 (7.6)
2017	888 (10)	94 (10.6)			338 (16)	32 (15.4)	1226 (8.7)	126 (9.9)
2018	1159 (13)	117 (13.1)	151 (4.9)	1 (0.6)	476 (22.5)	41 (19.7)	1786 (12.7)	159 (12.5)
2019	1301 (14.6)	147 (16.5)	728 (23.5)	17 (10)	401 (19)	45 (21.6)	2430 (17.2)	209 (16.5)
2020	998 (11.2)	118 (13.3)	659 (21.3)	35 (20.6)	230 (10.9)	37 (17.8)	1887 (13.4)	190 (15)
2021	1079 (12.1)	80 (9)	501 (16.2)	49 (28.8)	134 (6.3)	3 (1.4)	1714 (12.2)	132 (10.4)
2022	767 (8.6)	106 (11.9)	473 (15.3)	33 (19.4)	77 (3.6)	11 (5.3)	1317 (9.3)	150 (11.8)
2023	699 (7.9)	72 (8.1)	585 (18.9)	35 (20.6)	67 (3.2)	6 (2.9)	1351 (9.6)	113 (8.9)

Abbreviations: *AE*, adverse event; *B*, binimetinib; *BRAFi*, BRAF inhibitor; *cAE*, cardiovascular adverse event; *C*, cobimetinib; *D*, dabrafenib; *E*, encorafenib; *IME*, Important Medical Event; *Kg*, kilograms; *MEKi*, MEK inhibitor; *Q1*, quartile 1; *Q3*, quartile 3; *T*, trametinib; *V*, vemurafenib.

Table S3. Characteristics of AE reports for BRAFi and MEKi as monotherapy.

**Characteristic**	**V**		**C**		**D**		**T**		**E**		**B**		**Monotherapy**	
	**All AEs (*n* = 2174)**	**cAEs (*n* = 180)**	**All AEs (*n* = 263)**	**cAEs (*n* = 31)**	**All AEs (*n* = 852)**	**cAEs (*n* = 46)**	**All AEs (*n* = 695)**	**cAEs (*n* = 47)**	**All AEs (*n* = 122)**	**cAEs (*n* = 7)**	**All AEs (*n* = 161)**	**cAEs (*n* = 12)**	**All AEs (*n* = 4267)**	**cAEs (*n* = 323)**
Age (years), median (Q1-Q3)	60 (49-70)	65 (58-74)	66 (57-72)	69 (61-72)	61 (51-70)	69 (58-72)	64 (53-71)	64 (51-71)	58 (46-69)	77 (63-79)	67 (58-76)	55 (35-65)	62 (51-71)	65 (58-74)
Age group, *n* (%)														
Neonate							3 (0.4)						3 (0.1)	
Child	2 (0.1)				3 (0.4)		9 (1.3)						14 (0.3)	
Adolescent	2 (0.1)				4 (0.5)	2 (4.3)	4 (0.6)	3 (6.4)					10 (0.2)	5 (1.5)
Adult	879 (40.4)	65 (36.1)	99 (37.6)	13 (41.9)	325 (38.1)	15 (32.6)	239 (34.4)	21 (44.7)	53 (43.4)	2 (28.6)	38 (23.6)	5 (41.7)	1633 (38.3)	121 (37.5)
Elderly	592 (27.2)	84 (46.7)	112 (42.6)	16 (51.6)	225 (26.4)	23 (50)	213 (30.6)	15 (31.9)	36 (29.5)	4 (57.1)	55 (34.2)	4 (33.3)	1233 (28.9)	146 (45.2)
Not available	699 (32.2)	31 (17.2)	52 (19.8)	2 (6.5)	295 (34.6)	6 (13)	227 (32.7)	8 (17)	33 (27)	1 (14.3)	68 (42.2)	3 (25)	1374 (32.2)	51 (15.8)
Sex, *n* (%)														
Female	884 (40.7)	65 (36.1)	90 (34.2)	14 (45.2)	359 (42.1)	23 (50)	302 (43.5)	25 (53.2)	60 (49.2)	5 (71.4)	34 (21.1)	2 (16.7)	1729 (40.5)	134 (41.5)
Male	1159 (53.3)	103 (57.2)	169 (64.3)	17 (54.8)	445 (52.2)	23 (50)	361 (51.9)	20 (42.6)	48 (39.3)	1 (14.3)	84 (52.2)	7 (58.3)	2266 (53.1)	171 (52.9)
Not available	131 (6)	12 (6.7)	4 (1.5)		48 (5.6)		32 (4.6)	2 (4.3)	14 (11.5)	1 (14.3)	43 (26.7)	3 (25)	272 (6.4)	18 (5.6)
Weight (Kg), median (Q1-Q3)	73 (60-85)	73 (61-83)	76 (65-86)	80 (66-88)	75 (61-90)	61 (58-75)	75 (60-91)	65 (59-84)	75 (65-82)	77 (77-77)	81 (71-92)	104 (98-109)	74 (62-87)	72 (59-86)
Type of reporter, *n* (%)														
Consumer	597 (27.5)	12 (6.7)	54 (20.5)	1 (3.2)	249 (29.2)	5 (10.9)	205 (29.5)	10 (21.3)	35 (28.7)	4 (57.1)	71 (44.1)	3 (25)	1211 (28.4)	35 (10.8)
Physician	1168 (53.7)	148 (82.2)	156 (59.3)	29 (93.5)	275 (32.3)	23 (50)	230 (33.1)	22 (46.8)	28 (23)	2 (28.6)	30 (18.6)	1 (8.3)	1887 (44.2)	225 (69.7)
Pharmacist	137 (6.3)	4 (2.2)	31 (11.8)	1 (3.2)	96 (11.3)	3 (6.5)	77 (11.1)	2 (4.3)	12 (9.8)		7 (4.3)		360 (8.4)	10 (3.1)
Health-professional	25 (1.1)		9 (3.4)		63 (7.4)	2 (4.3)	77 (11.1)	6 (12.8)	28 (23)	1 (14.3)	35 (21.7)	7 (58.3)	237 (5.6)	16 (5)
Other health-professional	235 (10.8)	16 (8.9)	9 (3.4)		132 (15.5)	12 (26.1)	64 (9.2)	6 (12.8)	1 (0.8)		2 (1.2)		443 (10.4)	34 (10.5)
Not available	12 (0.6)		4 (1.5)		37 (4.3)	1 (2.2)	42 (6)	1 (2.1)	18 (14.8)		16 (9.9)	1 (8.3)	129 (3)	3 (0.9)
Outcome, *n* (%)														
Death	262 (12.1)	16 (8.9)	30 (11.4)	7 (22.6)	140 (16.4)	4 (8.7)	121 (17.4)	4 (8.5)	27 (22.1)	1 (14.3)	35 (21.7)	1 (8.3)	615 (14.4)	33 (10.2)
Disability	35 (1.6)	3 (1.7)	4 (1.5)		11 (1.3)		5 (0.7)				2 (1.2)		57 (1.3)	3 (0.9)
Life-threatening	47 (2.2)	6 (3.3)	3 (1.1)	1 (3.2)	13 (1.5)	3 (6.5)	4 (0.6)	2 (4.3)	1 (0.8)		4 (2.5)		72 (1.7)	12 (3.7)
Hospitalization - initial or prolonged	513 (23.6)	79 (43.9)	132 (50.2)	19 (61.3)	179 (21)	25 (54.3)	199 (28.6)	18 (38.3)	26 (21.3)	2 (28.6)	29 (18)	6 (50)	1078 (25.3)	149 (46.1)
Other serious (IME)	676 (31.1)	73 (40.6)	45 (17.1)	3 (9.7)	297 (34.9)	14 (30.4)	165 (23.7)	19 (40.4)	23 (18.9)	4 (57.1)	26 (16.1)	4 (33.3)	1232 (28.9)	117 (36.2)
Required intervention to prevent permanent impairment/damage							1 (0.1)	1 (2.1)					1 (<0.1)	1 (0.3)
Not available	641 (29.5)	3 (1.7)	49 (18.6)	1 (3.2)	212 (24.9)		200 (28.8)	3 (6.4)	45 (36.9)		65 (40.4)	1 (8.3)	1212 (28.4)	8 (2.5)
Reporter country, *n* (%)														
Africa	22 (1)		1 (0.4)		1 (0.1)		1 (0.1)						25 (0.6)	
Asia	237 (10.9)	36 (20)	22 (8.4)	2 (6.5)	34 (4)	4 (8.7)	13 (1.9)		2 (1.6)		1 (0.6)		309 (7.2)	42 (13)

Europe	737 (33.9)	100 (55.6)	123 (46.8)	25 (80.6)	247 (29)	29 (63)	186 (26.8)	17 (36.2)	15 (12.3)	2 (28.6)	15 (9.3)	2 (16.7)	1323 (31)	175 (54.2)
North America	1093 (50.3)	33 (18.3)	104 (39.5)	1 (3.2)	476 (55.9)	8 (17.4)	415 (59.7)	28 (59.6)	104 (85.2)	5 (71.4)	145 (90.1)	10 (83.3)	2337 (54.8)	85 (26.3)
Central America	13 (0.6)	3 (1.7)			4 (0.5)								17 (0.4)	3 (0.9)
South America	43 (2)	2 (1.1)	3 (1.1)	2 (6.5)	6 (0.7)		8 (1.2)	2 (4.3)					60 (1.4)	6 (1.9)
Oceania	27 (1.2)	6 (3.3)	9 (3.4)	1 (3.2)	21 (2.5)	4 (8.7)	19 (2.7)						76 (1.8)	11 (3.4)
Not available	2 (0.1)		1 (0.4)		63 (7.4)	1 (2.2)	53 (7.6)		1 (0.8)				120 (2.8)	1 (0.3)
Year of reporting, *n* (%)														
2014	248 (11.4)	30 (16.7)			111 (13)	2 (4.3)	35 (5)	3 (6.4)					394 (9.2)	35 (10.8)
2015	593 (27.3)	59 (32.8)	1 (0.4)		205 (24.1)	7 (15.2)	104 (15)	12 (25.5)			1 (0.6)		904 (21.2)	78 (24.1)
2016	770 (35.4)	37 (20.6)	35 (13.3)	5 (16.1)	109 (12.8)	13 (28.3)	61 (8.8)						975 (22.8)	55 (17)
2017	167 (7.7)	21 (11.7)	33 (12.5)	2 (6.5)	100 (11.7)	7 (15.2)	52 (7.5)	4 (8.5)					352 (8.2)	34 (10.5)
2018	142 (6.5)	13 (7.2)	52 (19.8)	11 (35.5)	67 (7.9)	4 (8.7)	69 (9.9)	7 (14.9)	2 (1.6)		8 (5)		340 (8)	35 (10.8)
2019	79 (3.6)	13 (7.2)	69 (26.2)	10 (32.3)	75 (8.8)	5 (10.9)	61 (8.8)	5 (10.6)	8 (6.6)	1 (14.3)	20 (12.4)	1 (8.3)	312 (7.3)	35 (10.8)
2020	79 (3.6)	2 (1.1)	33 (12.5)	3 (9.7)	81 (9.5)	4 (8.7)	90 (12.9)	7 (14.9)	22 (18)		45 (28)	3 (25)	350 (8.2)	19 (5.9)
2021	39 (1.8)	5 (2.8)	14 (5.3)		43 (5)		72 (10.4)	5 (10.6)	26 (21.3)	3 (42.9)	36 (22.4)	1 (8.3)	230 (5.4)	14 (4.3)
2022	31 (1.4)		14 (5.3)		33 (3.9)	1 (2.2)	106 (15.3)	1 (2.1)	33 (27)	3 (42.9)	19 (11.8)	4 (33.3)	236 (5.5)	9 (2.8)
2023	26 (1.2)		12 (4.6)		28 (3.3)	3 (6.5)	45 (6.5)	3 (6.4)	31 (25.4)		32 (19.9)	3 (25)	174 (4.1)	9 (2.8)

Abbreviations: *AE*, adverse event; *B*, binimetinib; *BRAFi*, BRAF inhibitor; *cAE*, cardiovascular adverse event; *C*, cobimetinib; *D*, dabrafenib; *E*, encorafenib; *IME*, Important Medical Event; *Kg*, kilograms; *MEKi*, MEK inhibitor; *Q1*, quartile 1; *Q3*, quartile 3; *T*, trametinib; *V*, vemurafenib.

Table S4. Disproportionality analysis for SMQs of interest with BRAFi and MEKi in monotherapy.

Bradyarrhythmias (incl conduction defects and disorders of sinus node function)									
	V			D			C		
PT	N	ROR (95% CI)	Expected	N	ROR (95% CI)	Expected	N	ROR (95% CI)	Expected
Electrocardiogram QT prolonged	34	12.1 (8.62–16.99)	Yes	5	4.49 (1.86–10.82)	No	3	8.78 (2.81–27.4)	No
Atrioventricular block*	3	4.7 (1.51–14.6)	No						
Bundle branch block right*	5	16.29 (6.77–39.23)	No						

Cardiac failure												
	V			D			T			C		
PT	N	ROR (95% CI)	Expected	N	ROR (95% CI)	Expected	N	ROR (95% CI)	Expected	N	ROR (95% CI)	Expected
Cardiac failure	21	2.63 (1.71–4.04)	No	9	2.88 (1.49–5.55)	Yes	4	-		4	4.16 (1.55–11.17)	No
Cardiac failure acute										4	58.11 (21.64–156.09)	No
Cardiac failure congestive	5	-										
Pulmonary oedema	7	-										

Cardiomyopathy												
	V			D			T			C		
PT	N	ROR (95% CI)	Expected	N	ROR (95% CI)	Expected	N	ROR (95% CI)	Expected	N	ROR (95% CI)	Expected
**IME**												
Cardiotoxicity	6	14.84 (6.65–33.1)	No				4	31.02 (11.6–82.96)	No			
Hypertensive cardiomyopathy	3	127.96 (40.78–401.49)	No									
Ejection fraction decreased*	9	5.96 (3.09–11.47)	No	5	8.46 (3.51–20.37)	Yes	12	25.18 (14.23–44.58)	Yes	8	44.95 (22.23–90.88)	Yes
Stress cardiomyopathy*										5	135.57 (55.89–328.84)	Yes

Tachyarrhythmias (incl supraventricular and ventricular tachyarrhythmias)									
	V			D			T		
PT	N	ROR (95% CI)	Expected	N	ROR (95% CI)	Expected	N	ROR (95% CI)	Expected
Atrial fibrillation	10	-		3	-		7	2.14 (1.02–4.51)	No
Atrial flutter*	5	6.2 (2.58–14.92)	No						
Sinus tachycardia*	3	3.13 (1.01–9.7)	No						

Ischaemic heart disease		
	V	D

PT	N	ROR (95% CI)	Expected	N	ROR (95% CI)	Expected
Angina pectoris	5	-				
Acute coronary syndrome	3	4.24 (1.37–13.16)	No			
Acute myocardial infarction	3	-				
Myocardial infarction	13	-		4	-	
Coronary artery disease*	3	-				

Embolism and thrombotic events												
	V			D			T			C		
PT	N	ROR (95% CI)	Expected	N	ROR (95% CI)	Expected	N	ROR (95% CI)	Expected	N	ROR (95% CI)	Expected
Disseminated intravascular coagulation	3	3.27 (1.05–10.16)	No	3	8.37 (2.69–26.01)	No						
Cerebrovascular accident	10	-					4	-				
Hemiparesis	3	-										
Ischaemic stroke										7	33.98 (16.04–72.02)	No
Transient ischaemic attack	4	-										
Pulmonary embolism	9	-					3	-		3	-	
Deep vein thrombosis	4	-										
Embolism							4	17.76 (6.64–47.47)	No			
Thrombosis				4	-		4	-				

Abbreviations: *BRAFi*, BRAF inhibitor; *C*, cobimetinib; *CI*, confidence interval; *D*, dabrafenib; *MEKi*, MEK inhibitor; *PT*, Preferred Term; *ROR*, reporting odd ratio; *SMQ*, Standardized MedDRA Query; *T*, trametinib; *V*, vemurafenib.

*Preferred Term (PT) not included in the Important Medical Event (IME) list.

Table S5. Selected cardiovascular adverse events reported in main registration studies.

Study	BRAF/MEKi	Number of patients	Cardiovascular adverse events	Any grade<br>%<br>(n. of patients)	Grade≥3<br>%<br>(n. of patients)
COMBI-d (10)	Dabrafenib + Trametinib	209	- Hypertension<br>- Decreased ejection fraction<br>- Peripheral edema	22% (46)<br>4% (9)<br>14% (30)	4% (8)<br><1% (1)<br><1% (1)
	Dabrafenib	211	- Hypertension<br>- Decreased ejection fraction<br>- Peripheral edema	14% (29)<br>2% (5)<br>5% (10)	5% (10)<br><1% (1)<br><1% (1)
COMBI-v (13)	Dabrafenib + Trametinib	350	- Hypertension<br>- Decreased ejection fraction<br>- Peripheral edema	26% (92)<br>8% (29)<br>12% (42)	14% (48)<br>4% (13)<br><1% (1)
	Vemurafenib	349	- Hypertension<br>- Decreased ejection fraction<br>- Peripheral edema	24% (84)<br>-<br>10% (35)	9% (32)<br>-<br><1% (1)
coBRIM (9)	Cobimetinib + Vemurafenib	247	- Hypertension<br>- Decreased ejection fraction<br>- Peripheral edema<br>- QT prolongation<br>- Atrial fibrillation<br>- Venous thromboembolism	16% (39)<br>12% (29)<br>14% (34)<br>5% (11)<br>4% (9)<br>1% (3)	6% (15)<br>2% (5)<br>-<br>1% (3)<br>1% (3)<br><1% (2)
	Vemurafenib + Placebo	246	- Hypertension<br>- Decreased ejection fraction<br>- Peripheral edema	8% (20)<br>5% (13)<br>11% (28)	3% (7)<br>1% (3)<br><1% (1)
			- QT prolongation<br>- Atrial fibrillation<br>- Venous thromboembolism	5% (13)<br>1% (3)<br><1% (2)	1% (3)<br>-<br><1% (1)
COLUMBUS (11)	Encorafenib+ Binimetinib	192	- Hypertension<br>- Decreased ejection fraction<br>- Cardiac failure<br>- QT prolongation<br>- Atrial fibrillation<br>- Venous thrombosis<br>- Myocardial infarction<br>- Tachycardia	5% (10) (G1-2)<br>5% (9) (G1-2)<br>1% (1)<br>-<br>-<br>1%(1)<br>1% (1)<br>1% (1)	6% (11)<br>1% (2)<br>1% (1)<br>-<br>1% (1)<br>-<br>1% (1)<br>-
	Vemurafenib	186	- Hypertension<br>- Decreased ejection fraction<br>- Cardiac failure<br>- QT prolongation<br>- Atrial fibrillation	8% (15) (G1-2)<br>1% (1)<br>-<br>3%(6)<br>1%(2)	3% (6)<br>-<br>-<br>-<br>-

			- Venous thrombosis<br>- Myocardial infarction<br>- Tachycardia	-<br>-<br>3% (5)	-<br>-<br>1% (1)
	Encorafenib	192	- Hypertension<br>- Decreased ejection fraction<br>- Cardiac failure<br>- QT prolongation<br>- Atrial fibrillation<br>- Venous thrombosis<br>- Myocardial infarction<br>- Tachycardia	3% (5) (G1-2)<br>1% (2)<br>-<br>3%(6)<br>2%(3)<br>1%(2)<br>-<br>2%(3)	3% (5)<br>1% (2)<br>-<br>1%(1)<br>-<br>1%(1)<br>-<br>1% (2)
COLUMBUS (12)	Encorafenib+Binimetinib	192	- Hypertension<br>- Decreased ejection fraction<br>- Peripheral edema<br>- Cardiac failure	16.7% (32)<br>5.7% (11)<br>12.5% (24)<br>1% (2)	7.3% (14)<br>1% (2)<br>1.6% (3)<br>0.5% (1)
	Vemurafenib	186	- Hypertension<br>- Decreased ejection fraction<br>- Peripheral edema<br>- Cardiac failure	12.9% (24)<br>0.5% (1)<br>10.8% (20)<br>1.1% (2)	3.8% (7)<br>-<br>1.1% (2)<br>0.5% (1)
	Encorafenib	192	- Hypertension<br>- Decreased ejection fraction<br>- Peripheral edema<br>- Cardiac failure	6.3% (12)<br>2.1% (4)<br>9.4% (18)<br>-	3.6% (7)<br>1% (2)<br>-<br>-
COMBI-AD (14)	Dabrafenib + Trametinib	435	- Peripheral edema<br>- Hypertension	13% (58)<br>12% (51)	>1% (1)<br>6% (26)
	Placebo	432	- Peripheral edema<br>- Hypertension	5% (21)<br>9% (37)	-<br>2% (9)
